# Accurate reduction of medial arch fracture fragments in intertrochanteric fractures: A novel technical note

**DOI:** 10.3389/fsurg.2023.1140250

**Published:** 2023-02-27

**Authors:** Xiaodong Li, Chen Zhao, Guantong Sun, Pengcheng Liu, Jian Tang, Fei Yang, Xiaoqing Wang

**Affiliations:** ^1^ Department of Orthopedics, Shanghai Ninth People’s Hospital, School of Medicine, Shanghai Jiao Tong University, Shanghai, China; ^2^ Shanghai Key Laboratory of Orthopedic Implants, Department of Orthopedic Surgery, Shanghai Ninth People's Hospital, Shanghai Jiaotong University School of Medicine, Shanghai, China

**Keywords:** Intertrochanteric fractures, medial arch, right angle forceps, reduction techniques, enhanced recovery after surgery (ERAS)

## Abstract

Intramedullary fixation is currently used to stabilize intertrochanteric fractures. Surgical reduction of the medial arch cortex is crucial to achieve stabilization of the internal fixation system, however, it is challenging to perform. To ensure anatomical reduction, we developed a novel surgical technique to assist in achieving accurate and convenient reduction. In this technique, right-angle forceps were used to pry and reset medial arch cortex fragments *via* a mini-helical blade incision. Noteworthily, all patients who underwent this technique achieved anatomical reduction with reduced operation times and bleeding. Our article illustrates intraoperative reduction techniques and summarizes tips and tricks that may be beneficial and educative for orthopedists.

## 1. Introduction

The incidence of intertrochanteric fractures is increasing with population aging, and the one-year mortality of this condition ranging from 14% to 36% (1). Fractures in elderly individuals with osteoporosis may be caused by accidental falls or minor trauma, and are usually treated surgically by closed reduction and internal fixation (2). Even if intramedullary nailing is reliable for the treatment of intertrochanteric fractures, especially involving medial or lateral wall destruction, the reduction of the medial arch cortex by closed manipulation is challenging. Research has suggested that adequate reduction of medial arch fracture fragments is important to achieve postoperative stability and rehabilitation of intertrochanteric fractures (3–5). Furthermore, understanding the deforming forces acting on various fracture patterns and using proper surgical techniques is important to ensure construct stability.

Several studies have previously indicated that point reduction clamps or even cerclage wire could contribute to reduction of the medial arch cortex fragments in subtrochanteric fractures, while causing great disturbance to surrounding soft tissues (6–9). However, to date, few studies have investigated irreducible medial arch fracture fragment displacement in patients with intertrochanteric fractures. In the current study, we made a mini-helical blade incision to palpate the medial arch fracture fragments, and then used right-angle forceps to reduce the displaced fracture fragments. This surgical technique has two novel aspects. First, the right-angle forceps interfere less with the soft tissues around the fracture fragments to minimize intraoperative bleeding. Second, this technique can be easily performed by an assistant surgeon, saves operation time, and is conducive to postoperative rehabilitation. Here, we describe the surgical techniques in detail.

## 2. Methods

### 2.1. Patients

Between June 2021 and March 2022, we operated on ten consecutive patients with displaced intertrochanteric fractures. Nine patients were female, and one was male, with an average age of 83.9 years. According to the latest AO/OTA classification (2018 edition), the fractures were categorized as 31-A1.3 in three cases, 31-A2.2 in five cases, 31-A3.1 in one case, and 31-A3.3 in one case. None of the patients had any other concomitant injury.

### 2.2. Position placement

Under general anesthesia, the patients were placed in the supine position on a radiolucent traction table. Antibiotic prophylaxis was administered approximately 30 min prior to the surgical incision. The involved lower limb was stabilized securely in the traction boot, and the limb was placed at approximately 10–15° in adduction, while the uninjured extremity was placed in abduction and flexion positions to facilitate intraoperative fluoroscopy. The foot must be handled with proper care to avoid skin abrasions. In addition, the upper trunk should be angled opposite to the fractured side to prevent obstruction of the entry point and guidewire insertion.

### 2.3. Surgical technique

The injury-to-surgery interval was 2 days on average (range, 1.5–4 days), and we were able to manage the reduction of the medial arch fracture fragments quickly and accurately during the operation.

**Figure 1:**
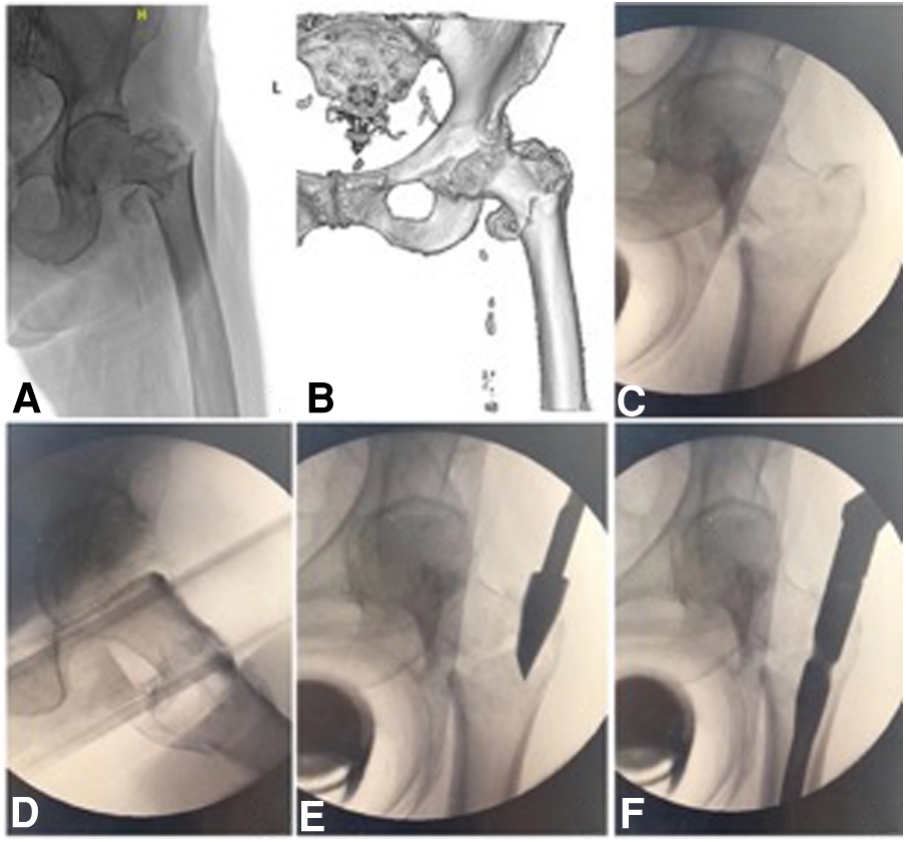
An 81-year-old woman with a comminuted intertrochanteric fracture, involving the medial arch and lateral wall was graded as AO/OTA 31-A2.2 (2018 edition). Preoperative x-ray (**A**) and CT scan (**B**) show the comminuted intertrochanteric fracture. (**C,D**), anteroposterior and lateral images show that the reduction was acceptable under axis traction. (**E,F**) The entry point was confirmed, and the displaced medial arch fragments developed after the femoral nail insertion.

**Figure 2:**
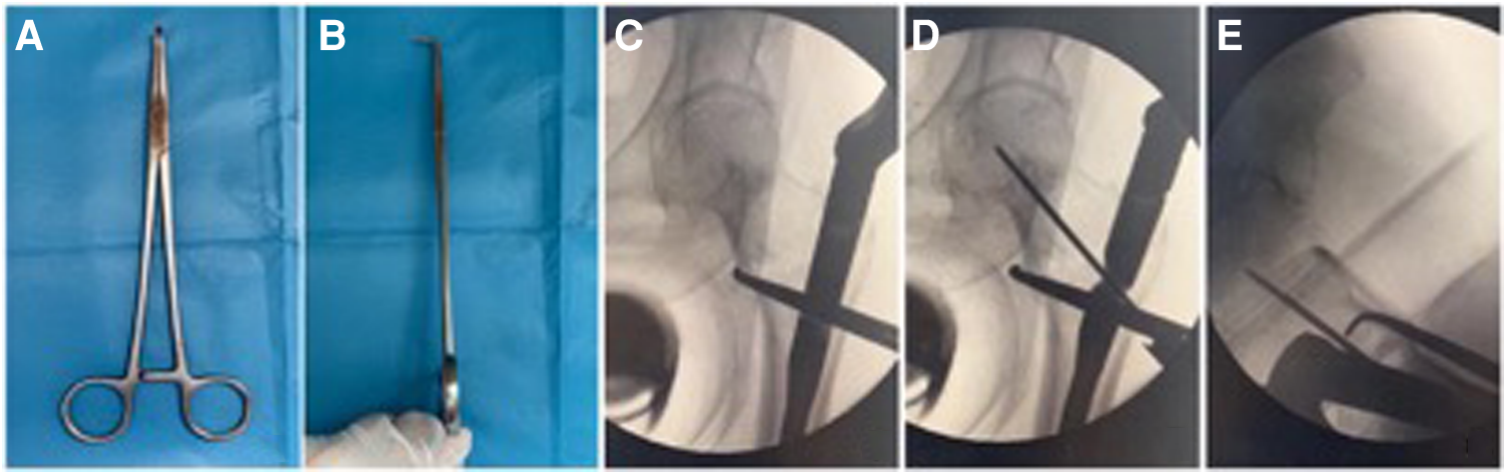
Right-angle forceps were used in the reduction of medial arch fragments. (**A,B**) shows the right-angle forceps. (**C,D**) The right-angle forceps were used to pry apart the medial arch fragments, and then the helical blade guide wire was inserted and confirmed under anteroposterior and lateral images.

**Figure 3:**
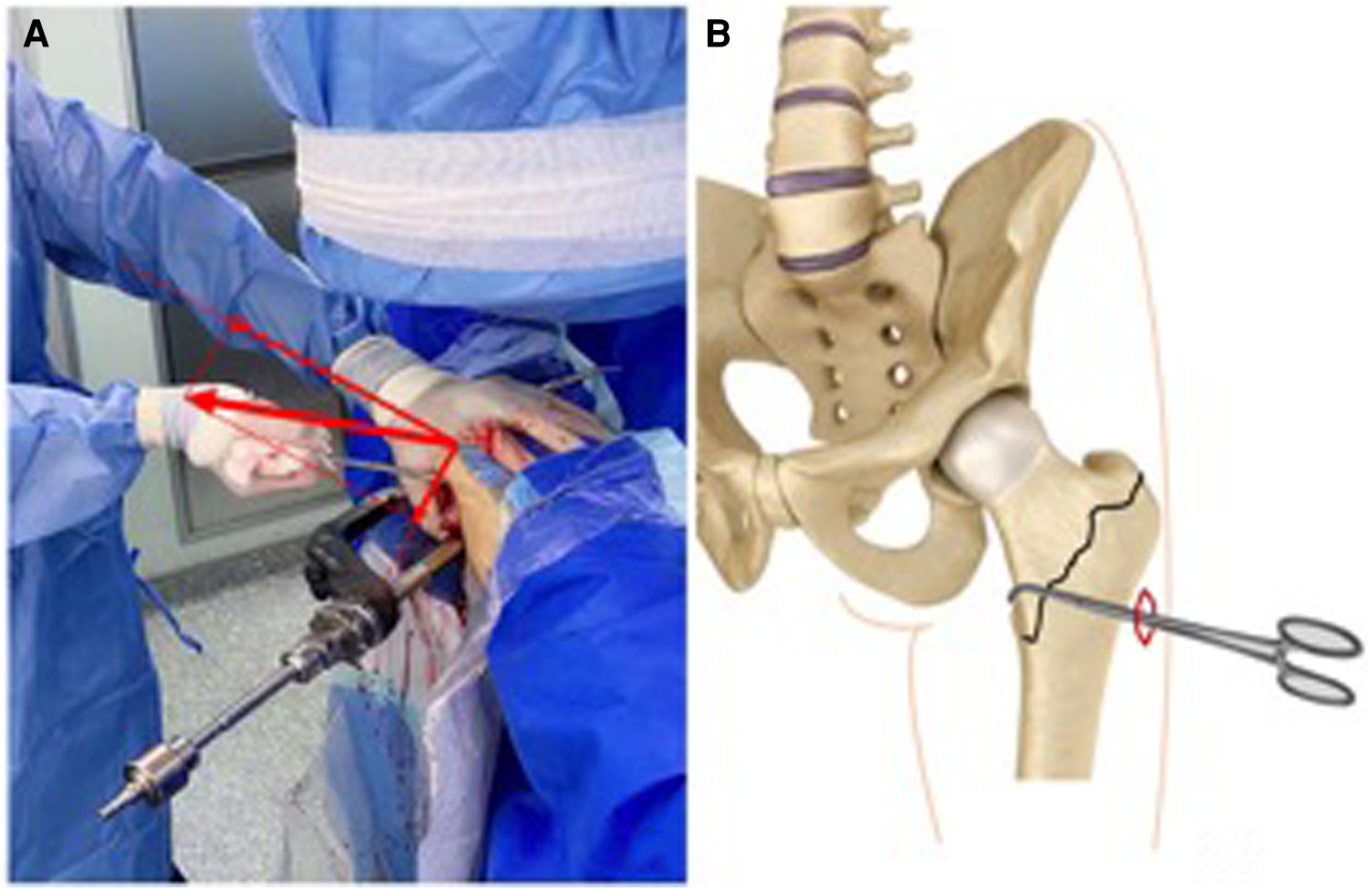
The minimally invasive reduction techniques were performed as follows: (**A**) the assistant surgeon palpated and pried the medial arch fragments. (**B**) Diagram of the minimally invasive reduction techniques: the right-angle forceps were passed anteriorly to assist the reduction of the medial arch fragments even the displaced lesser trochanter through the cephalomedullary incision.

**Figure 4:**
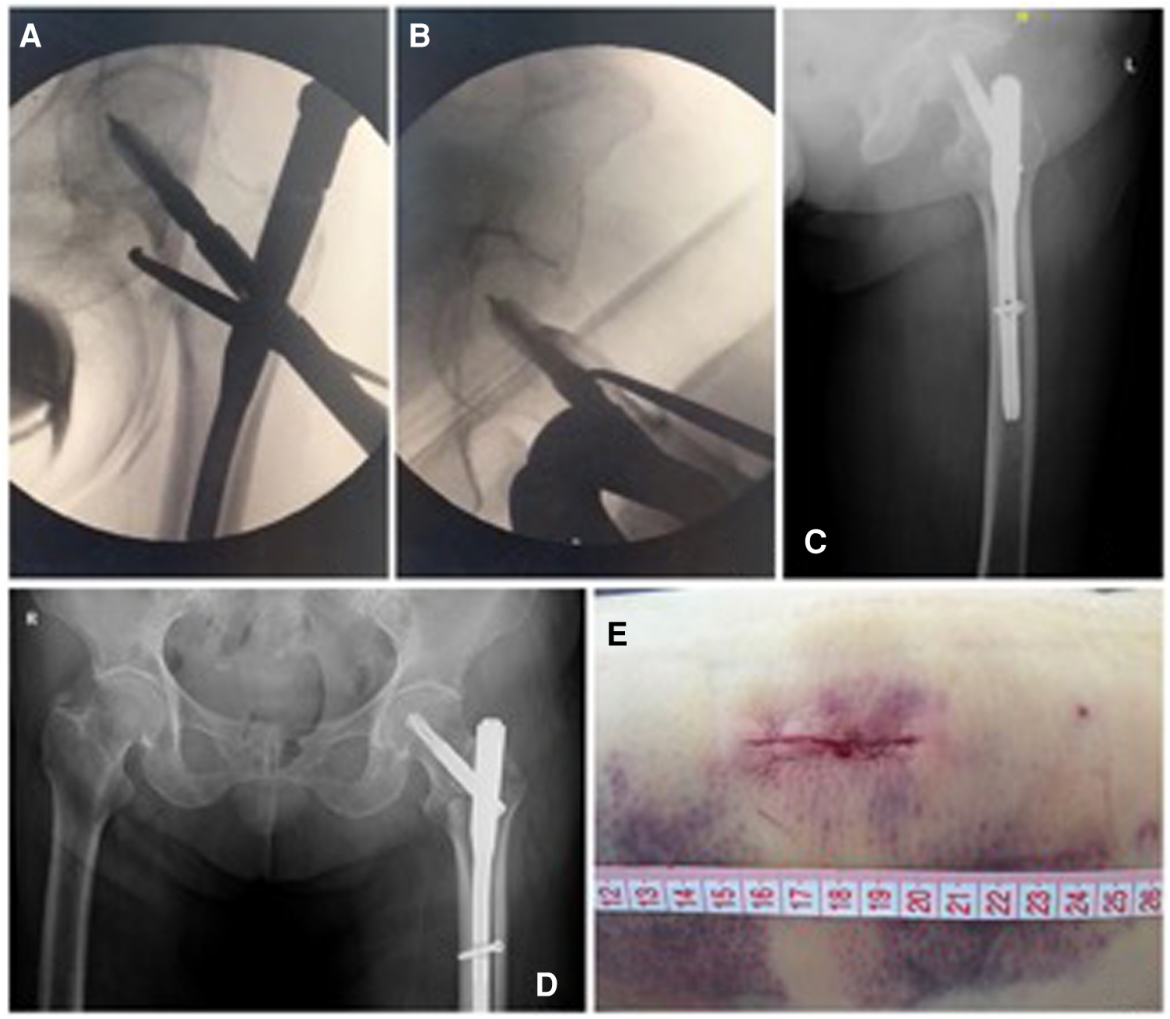
Routine maneuvers during nail fixation. (**A,B**) Anteroposterior and lateral views of the anatomical reduction and satisfactory fixation by PFNA. (**C,D**) Postoperative radiographs showing perfect fixation of the intertrochanteric fracture. (**E**) A minimal incision was used for reduction of the medial arch fragments and helical blade insertion.

After restoring the neck shaft and anteversion angles on the traction table, a lateral incision was made starting approximately 2 cm proximal to the greater trochanter along the axis of the femur. The optimal entry point was confirmed by the cannulated awl according to intraoperative fluoroscopy and placed slightly more medial to the tip of the greater trochanter in the anteroposterior view and in the center of the femoral trochanter in the lateral view (Figure 1). The guidewire was advanced from the greater trochanter to the distal femur fragments, and the final position was confirmed by fluoroscopy, as eliciting a reduction of medial arch fragments may not be ideal. Minimally invasive reduction techniques were recommended when a femoral intramedullary nail was inserted. We used right-angle forceps that passed the lesser trochanter anteriorly and palpated the medial arch fracture fragments to assist in the reduction of the medial arch cortex through the mini-helical blade incision (Figure 2). The assistant surgeon pried the medial arch fracture fragments, and subsequently inserted the helical blade guide wire. Fluoroscopy confirmed that this procedure was successful at achieving anatomical reduction of the medial arch cortex (Figure 3). The reduction techniques not only inflicted limited intrusion to the soft tissues around the fractures, but were also easily manipulated by surgeons with minimal experience. Furthermore, it could resist the wedge effect when a femoral intramedullary nail was inserted. After the intramedullary nail became statically locked, the wound was irrigated with saline and closed in layers (Figure 4), and a suction drain was deemed not necessary.

**Figure 5:**
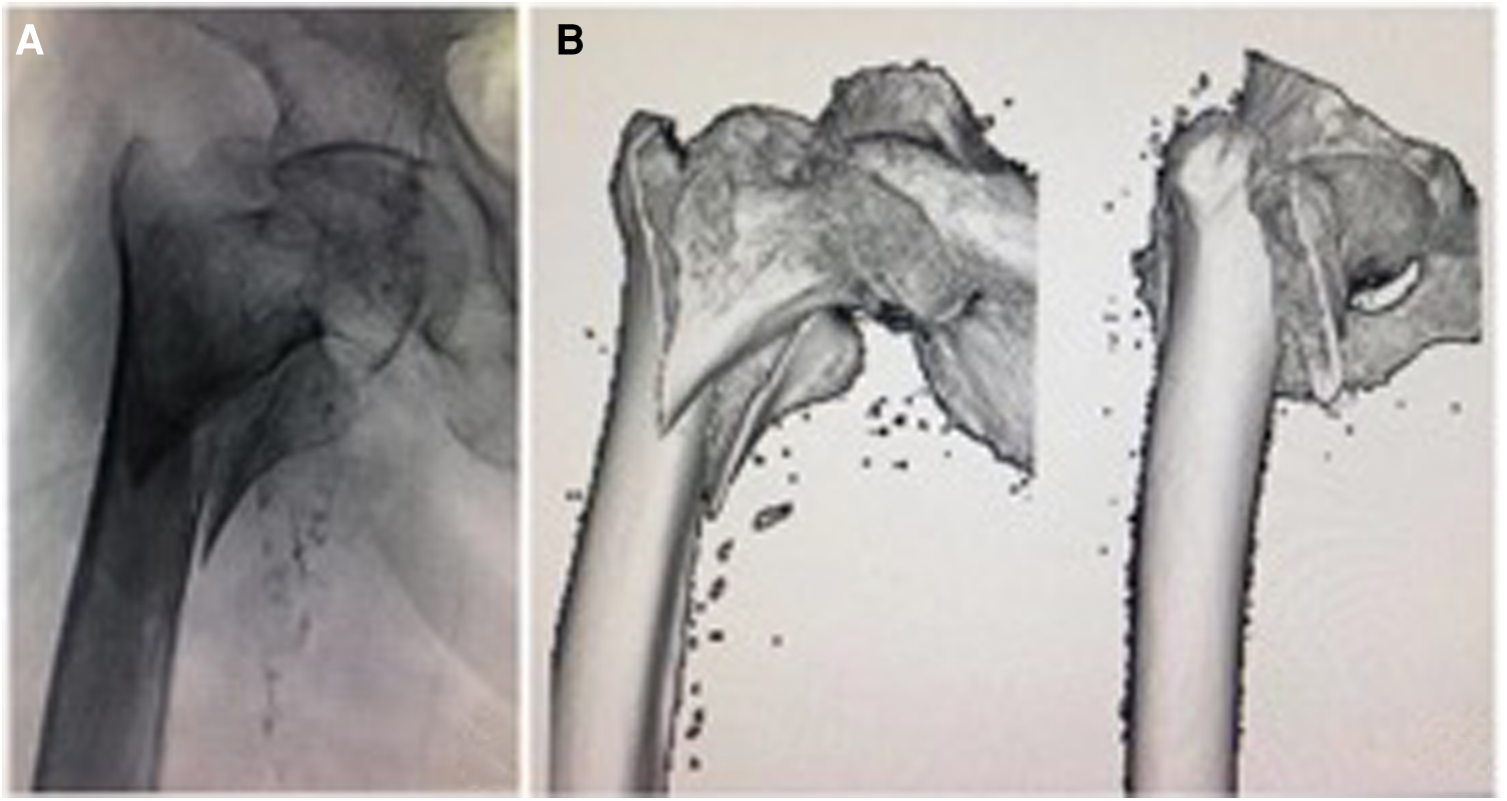
Intertrochanteric fracture in another patient, a 97-year-old woman, in which the medial arch was involved and displaced anteriorly. Preoperative x-ray (**A**) and CT scan (**B**) show that the intertrochanteric fracture and the medial arch fragment were displaced anteriorly.

**Figure 6:**
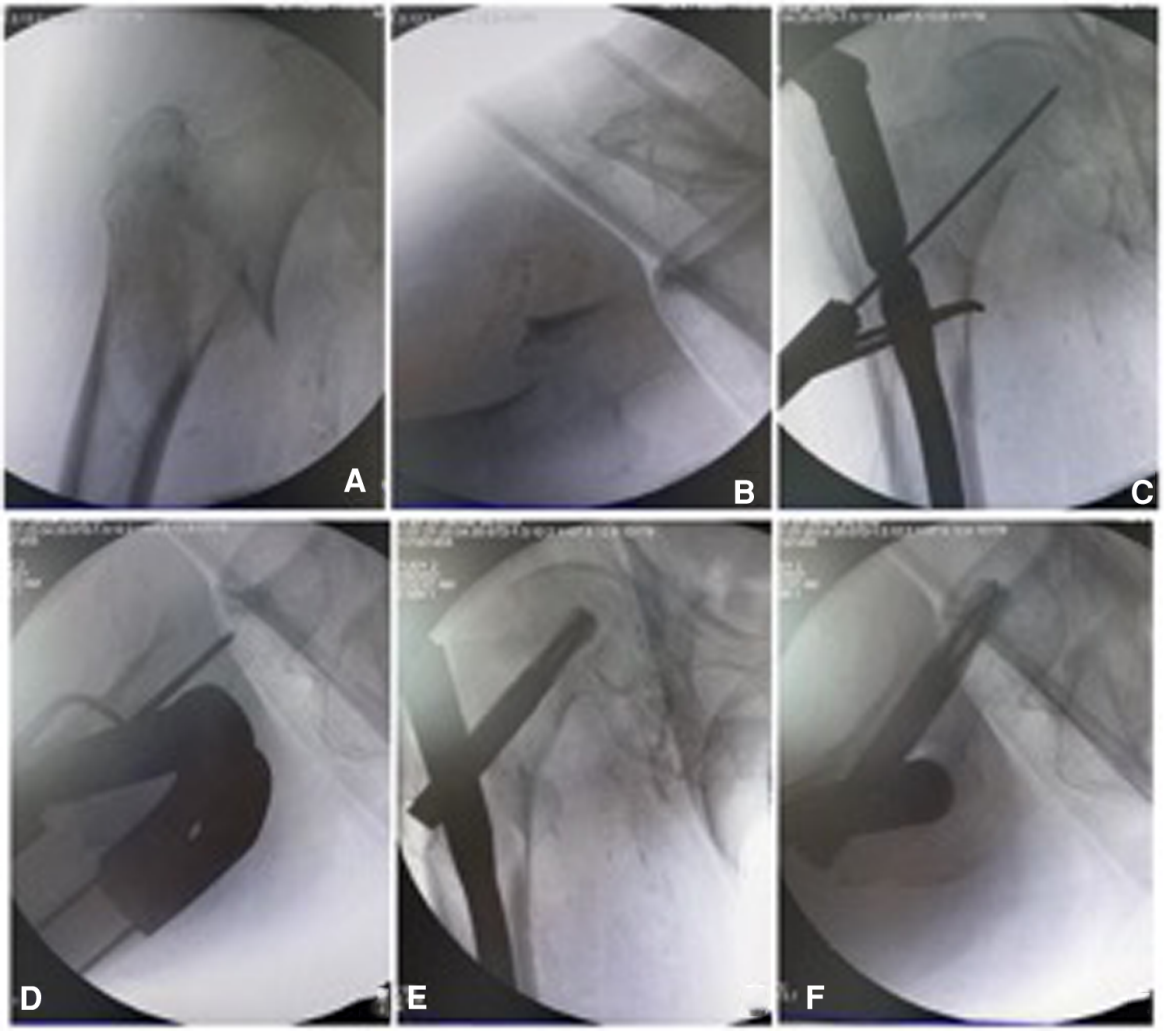
The right-angle forceps was used for reduction of the medial arch fragments. (**A,B**) Under axis traction, anteroposterior and lateral images show the medial arch fragment displaced anteromedially. (**C,D**) the right-angle forceps were used to pry the medial arch fragment, which was confirmed under anteroposterior and lateral images. (**E,F**) The helical blade was inserted and anatomical reduction of the medial arch was achieved.

In addition to the reduction of medial displaced fracture fragments, anteriorly displaced fragments could be reset effectively using our reduction techniques (Figure 5). By pressing the anteriorly displaced lesser trochanter fragment and resisting the wedge effect with right-angle forceps, the reduction of the medial arch fracture fragments could be successfully retained (Figure 6). Importantly, the entire operation time was dramatically reduced, which may guarantee the implementation of enhanced recovery after surgery (ERAS) and reduce the risk of postoperative complications. All patients had an average blood loss of 80 ml, and the mean operation time to manage these intertrochanteric fractures was 35 min (range, 30–75 min). Conventionally, low molecular weight heparin was applied postoperatively to prevent deep vein thrombosis.

## 3. Results

After applying the reduction techniques of right-angle forceps, all patients achieved anatomical reduction, especially of the displaced medial arch cortex, with reduced operation time and blood loss. No complications, such as infection, delayed union or nonunion, implant breakage, or helical blade cutoff, were observed in any of the patients, and routine follow-up was performed until fracture healing. (Table 1) The average follow-up time was 8.4 months. Finally, all patients returned to full weight-bearing when an obvious callus was found.

**Table 1:** Patients demographics and results.

Case	Sex	Age	Operation time (min)	Blood loss (ml)	Complications	Union time (weeks)	Follow-up months
1	F	90	30	55	No	8	6
2	F	86	75	120	No	10	11
3	M	77	30	85	No	11	7
4	F	90	32	95	No	8	10
5	F	97	30	70	No	13	6
6	F	80	30	90	No	10	8
7	F	70	31	65	No	12	8
8	F	81	33	45	No	9	6
9	F	91	32	100	No	11	12
10	F	78	30	75	No	14	10

## 4. Discussion

Consensus on reduction and intramedullary nail fixation has become the optimum treatment for unstable intertrochanteric fractures among orthopedic surgeons, and anatomical reduction of fractures is significant following fixation and rehabilitation (10). The integrity of the medial arch is an important factor for the stability and healing of intertrochanteric fractures (5, 11, 12). Anatomical reduction of medial arch fracture fragments is of critical importance in intertrochanteric fractures. Many surgical reduction techniques have been applied to achieve satisfactory reduction of irreducible reverse intertrochanteric or subtrochanteric fractures, such as point reduction clamps, bone hooks, and Hohmann retractors (7, 13, 14). However, the limitations of these studies should be noted. These surgical reduction techniques might require open reduction or extended skin incision and can cause significant damage to the surrounding soft tissues, resulting in increased bleeding and operation time (15). In addition, cerclage was used, followed by disruption of the regional blood circulation and delayed fracture healing (16). Therefore, we developed a novel surgical technique to achieve reduction of the medial arch fracture fragments, even the lesser trochanter, using right-angle forceps through the mini helical blade incision without additional damage.

The morphological character of most medial arch fragments is the femoral neck connecting with the lesser trochanter along the sagittal plane, such as AO/OTA 31-A1.3 and 31-A2.3(2007 edition), resulting in a loss of anteromedial support, which is consistent with other research results (17). The lesser trochanter fracture fragment is displaced anteromedially under the traction of the iliacus, and it is difficult to achieve anatomical reduction by relying solely on traction and internal rotation of the lower limbs. Therefore, our novel reduction technique is suitable for these types of fractures. Crucially, the reduction techniques not only requires little intrusion into soft tissues around the fracture, but can also be easily performed by beginners. Moreover, it could resist the wedge effect when a femoral intramedullary nail was inserted. Research shows that prolonged operative duration is associated with postoperative complications, such as surgical site infection, urinary tract infection, pneumonia, and cardiac complications (18, 19). Importantly, our novel reduction techniques could reduce operation time, and be conducive to rapid postoperative recovery.

In addition to restoring fracture fragments satisfactorily, minimizing detrimental effects and complications has always been the goal of orthopedic surgeons. Although the number of patients treated with our novel surgical techniques in this study was small, we nevertheless found that this novel procedure guaranteed reduction of medial arch fragments, even in the lesser trochanter, with rapid recovery observed during follow-up. This novel surgical technique is simple and can be directly applied in the care of difficult intertrochanteric fractures, especially the reduction of medial arch fracture fragments. However, the limitations of our study should be noted as well. First, the number of relevant cases is small due to the outbreak of COVID-19. Second, it may be more convincing if more surgical teams use the novel surgical technique. Even so, we believe the novel surgical technique is simple and good in reduction of the medial arch fracture fragments.

## Data Availability

The raw data supporting the conclusions of this article will be made available by the authors, without undue reservation.
